# *Capnocytophaga canimorsus* Infection in a 38-Year-Old Male after a Dog Bite

**DOI:** 10.1155/2023/9917898

**Published:** 2023-10-16

**Authors:** Ahmad Ahsen, Philip Korsun, Fadi Albahra, Ranjit Nair, Zain Tariq

**Affiliations:** ^1^Department of Internal Medicine, Medical City Fort Worth, Fort Worth, TX, USA; ^2^Department of Critical Care, Medical City Fort Worth, Fort Worth, TX, USA; ^3^Department of Infectious Disease, Medical City Fort Worth, Fort Worth, TX, USA

## Abstract

Here, we present a unique case of a 38-year-old male with a history of alcohol use disorder and multiple sexual partners, who presented with fulminant sepsis with shock, multiorgan failure, and livedo racemosa after a dog bite the week prior. The patient was intubated on arrival and was started on vasopressors and antibiotics. Eventually, the patient's clinical status improved, and he was transferred out of the intensive care unit. Blood cultures tested positive for oxidase-positive Gram-negative rods two days after collection, and species identification showed *Capnocytophaga canimorsus*.

## 1. Introduction

*Capnocytophaga canimorsus* is a rare but deadly cause of infection from contact with a dog, including, but not limited, to bites [1–5]. Symptoms include, but are not limited to, disseminated purpura, disseminated intravascular coagulation (DIC), septic shock, bacteremia, multiorgan failure, altered mental status, and meningitis [1–8]. People at risk are immunocompromised individuals, especially those with asplenia, cirrhosis, and heavy alcohol use [6]. *Capnocytophaga* should be suspected if a patient presents with multiorgan failure after a dog bite, especially if they present with livedo racemosa [1–12].

## 2. Case Report

This patient is a 38-year-old male who was admitted for rapid onset acute hypoxic respiratory failure, requiring intubation. He had a past medical history of alcohol use disorder and multiple sexual partners. On the day of admission, he had nausea and vomiting as well as multiple episodes of diarrhea. He was cyanotic and had shortness of breath.

**Figure 1:**
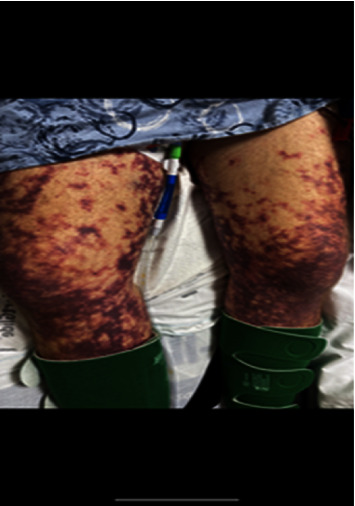
Livedo racemosa is present on the patient's legs.

**Figure 2:**
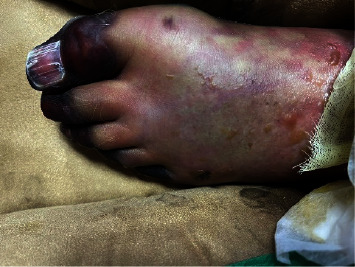
The patient had distal necrosis of the toes.

On examination, he had livedo racemosa on both lower extremities (Figure 1). He also had gangrene on his toes (Figure 2). He was in cardiogenic and septic shock (Table 1). Further workup showed acute liver failure and acute tubular necrosis requiring continuous renal replacement therapy (CRRT), DIC, and blood loss anemia (Table 2). He was treated supportively for DIC.

**Figure 3:**
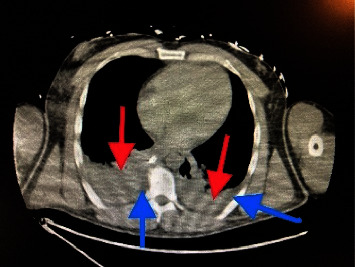
The red lines are pointing towards bilateral pleural effusions. The blue lines are pointing towards hypointense densities, which indicate bilateral atelectasis.

**Figure 4:**
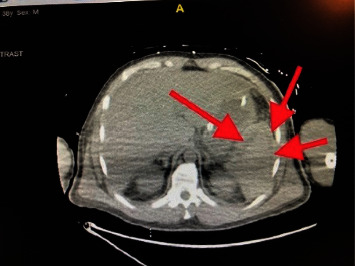
A subcapsular hematoma is present on the anterolateral aspects of the spleen.

**Figure 5:**
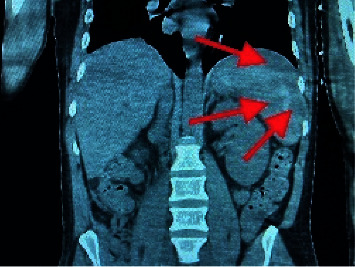
The hypointense lesions showing multiple splenic infarcts.

**Figure 6:**
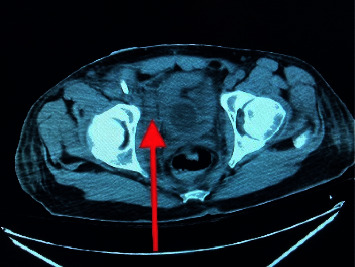
Right extraperitoneal hematoma.

**Figure 7:**
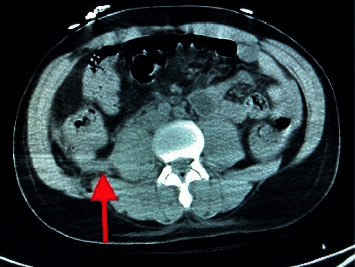
Right retroperitoneal hematoma.

The computerized tomography (CT) chest, abdomen, and pelvis showed severe right lower lobe and moderate left lower lobe dependent atelectasis with possible consolidation (Figure 3), subcapsular splenic hematoma, and multiple splenic infarcts (Figures 4 and 5). There was also hemorrhage in the right extraperitoneal space (Figure 6) extending into the right inferior retroperitoneal space (4.3 × 7.8 × 12.9 cm) (Figure 7). Abdominal ultrasound showed increased liver echogenicity with hepatomegaly (liver span 20.6 cm). Echocardiogram showed an ejection fraction of 35–40%.

**Figure 8:**
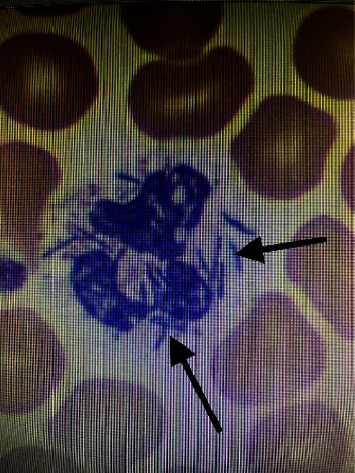
*Capnocytophaga canimorsus* is a Gram-negative rod, seen in the polymorphonuclear leukocytes on this image.

Initial treatment included vancomycin, meropenem, doxycycline, and ampicillin-sulbactam for broad spectrum coverage. Patient's sepsis and lactic acidosis continued to worsen despite treatment. Then, micafungin was initiated to cover for fungal etiologies. Then, blood cultures grew Gram-negative oxidase-positive rods. Matrix-assisted laser desorption/ionization time-of-flight mass spectrometry (MALDI-TOF MS) was used to identify the bacteria as *Capnocytophaga canimorsus*. Furthermore, peripheral blood smear showed intracellular rod-shaped bacteria (Figure 8). Meropenem was continued while all the other antimicrobials were stopped. Clindamycin was added. The patient was weaned off vasopressors and clinically improved.

## 3. Discussion and Review of the Literature

In this section, we will discuss the risk factors for infection with *Capnocytophaga* and its association with livedo racemosa [9, 10]. We will also discuss diagnostic modalities.

In a study of thirty-nine cases of *Capnocytophaga* in Denmark, six people had a history of alcohol use disorder, like our patient [2]. Alcohol abuse, cirrhosis, and asplenia are risk factors for infection with *Capnocytophaga canimorsus* [2, 3]. Our patient had no risk factors for being immunocompromised other than his history of alcohol use disorder. None of the literature reviewed suggested that having multiple sexual partners increased risk of infection.

Another interesting, though rare, feature of *Capnocytophaga* is its association with livedo racemosa. *Capnocytophaga* infection should be suspected in a patient who presents with signs of infection after a recent dog bite and has livedo racemosa, which can present before multiorgan failure [9, 10].

Livedo racemosa can be confused with livedo reticularis since they look similar. Both types of rashes present as violaceous net-like patterns on the skin [11]. In contrast to livedo reticularis, however, livedo racemosa is segmented, irregular, circular, and broken [11]. Skin is one of the earliest organs *Capnocytophaga* seems to infect [9]. Moreover, livedo racemosa is irreversible in warm temperatures, while livedo reticularis is only present in cold temperatures [9]. Unlike livedo reticularis, livedo racemosa is not only present on the extremities but also on the buttocks and trunk [11].

In contrast to livedo racemosa and livedo reticularis, purpura fulminans presents with central areas of irregular hemorrhagic necrosis and is nonblanchable [13]. In the setting of *Capnocytophaga* infection, sometimes livedo racemosa can precede purpura fulminans and multiorgan failure [9, 10].

Identifying livedo racemosa can assist with early diagnosis of infection with *Capnocytophaga*, leading to early management. Giving antibiotics early has been shown by an observation study to improve outcomes [1]. As mentioned earlier, we utilized meropenem and clindamycin. Beta-lactamase- and carbapenemase-resistant strains are susceptible to clindamycin [6, 14]. Rapidly diagnosing *Capnocytophaga* is difficult since it requires a longer time to grow on blood cultures, averaging about six days to test positive [7]. These bacteria appear as long fusiform Gram-negative rods on blood cultures and evade the immune system by inhibiting phagocytosis by macrophages and killing by polymorphonuclear leukocytes [15–20].

Polymerase chain reaction (PCR) and nanopore sequencing in whole blood may be other modalities that can be utilized for more rapid diagnosis in the future; they are not available for clinical use yet [21–23]. Peripheral blood smear is another way to expedite diagnosis, as it shows *Capnocytophaga* in the polymorphonuclear leukocytes [24].

## 4. Conclusion

*Capnocytophaga canimorsus* should be high on the differential when a person develops severe sepsis with multiorgan dysfunction and DIC after a dog bite, especially if he is immunocompromised [1–8]. It is important to be able to identify livedo racemosa and be able to distinguish it from livedo reticularis and purpura fulminans [11, 13]. Livedo racemosa, when present on exam, can provide a strong clue that the patient is infected with *Capnocytophaga*.

Finding Gram-negative rods intracellularly in the peripheral blood smear is yet another clue to help identify *Capnocytophaga* [24]. Moreover, blood PCR can possibly identify DNA sequences belonging to *Capnocytophaga*; this could possibly expedite diagnosis if they were clinically available [21–23]. The peripheral blood smears and CT scan were taken from hospital records of the patient. The photographs show the patient's extremities.

## Figures and Tables

**Table 1:** Initial vitals.

Vital signs (references)	Patient's vitals
Temperature (98.6 F)	101.5
Heart rate (60–100)	134
Blood pressure (120/80 mmHg)	61/36
Oxygen saturation (>92%)	42
Respiration rate (12–16 breaths/minute)	49

**Table 2:** Lab values.

Patient's values	Lab (reference)
39.4	PT (11 to 13.5 seconds)
286	PTT (25–35 seconds)
80	Fibrinogen (200–400 mg/dL)
>128,000	D-dimer (220–500 ng/mL)
20.10	C-reactive protein (<0.29 mg/dL)
11.9	White blood cell (4.5–11 K/MM^3^)
17	Platelet (150–450 K/MM^3^)
15	Bicarbonate (22–29 mmol/L)
93	Chloride (96–106 mmol/L)
10.7	Phosphorus (2.8–4.5 mg/dL)
4.9	Creatinine (0.74–1.35 mg/dL)
14,206	AST (8–33 unit/L)
4029	ALT (4–36 unit/L)
8.2	Total bilirubin (0.1–1.2 mg/dL)
4.6	Direct bilirubin (<0.3 mg/dL)
4167	Troponin (<78 ng/L)
20.5	Lactic acid (<2 mmol/L)
7.085	Arterial blood gas pH (7.35–7.45)
63.8	Arterial blood gas partial pressure CO_2_(35–45 mmHg)
54	Arterial blood gas partial pressure O_2_(75–100 mmHg)

## Data Availability

The data supporting the results of the study include the patient's labs, vitals, imaging, and pictures of his symptoms. The source of the data for this study cannot be publicly accessed as it would reveal unique patient identifier information, which would be illegal and unethical.
